# The modulation of neural insular activity by a brain computer interface differentially affects pain discrimination

**DOI:** 10.1038/s41598-021-89206-3

**Published:** 2021-05-07

**Authors:** Philipp Taesler, Michael Rose

**Affiliations:** Department of Systems Neuroscience, University Medical Center Hamburg-Eppendorf, Martinistr. 52, W34, 20251 Hamburg, Germany

**Keywords:** Neuroscience, Psychology

## Abstract

The experience of pain is generated by activations throughout a complex pain network with the insular cortex as a central processing area. The state of ongoing oscillatory activity can influence subsequent processing throughout this network. In particular the ongoing theta-band power can be relevant for later pain processing, however a direct functional relation to post-stimulus processing or behaviour is missing. Here, we used a non-invasive brain–computer interface to either increase or decrease ongoing theta-band power originating in the insular cortex. Our results show a differential modulation of oscillatory power and even more important a transfer to independently measured pain processing and sensation. Pain evoked neural power and subjective pain discrimination were differentially affected by the induced modulations of the oscillatory state. The results demonstrate a functional relevance of insular based theta-band oscillatory states for the processing and subjective discrimination of nociceptive stimuli and offer the perspective for clinical applications.

## Introduction

Whether a given (i.e. nociceptive) stimulus is perceived as painful or not depends on various factors, from stimulus intensity through perceptual sensitivity and attention to mental states such as expectation^1,2^. In recent years, research has converged on a complex network involved in nociceptive processing above the brainstem. This network is usually reported to include S1, S2, Insular Cortex (IC), Anterior Cingulate, Cortex, Prefrontal Cortex and the Thalamus^3^. The interaction within this network is in some parts overlapping with the processing of somatosensory, non-painful stimulation, rendering it difficult to disentangle activations in experimental paradigms^4,5^. Most of the network nodes, specifically the IC, have also been implicated in other, non-pain related networks, such as for salience^6–8^ and interoception^9^.

For pain, the posterior part of the insula is associated with an early, specific nociceptive processing stage, whereas the anterior part is involved in a later, more cognitively modulated integrative process, involved in salience allocation and a conscious percept^10–13^. Recent observations on the time course of insula activation in pain^14^ as well as previous findings on functional interconnections between anterior and posterior insula^15,16^ suggest, that it is an important integrative hub in the pain processing network^9^ with a functional dissociation between salience and more specific pain processing^17^. In particular the function of the anterior insula reflects internal network states, which can modulate salience and the susceptibility to pain states^18,19^. These network states can be represented in ongoing activity prior to stimulus onset and may be accessible to voluntary influence. Only few studies have examined the anticipation phase preceding the pain stimulus to address the initiation of pain-related expectation. For example it was shown that the activity of the DLPFC and OFC during the anticipation phase correlated with the observed placebo effect during subsequent heat pain^20^. The importance of pre-stimulus activity for pain processing was further demonstrated in an fMRI study, that showed that the functional connectivity of the anterior insula with the brainstem prior to stimulus presentation determines whether a noxious event is perceived as painful^21^. Further, it was shown, that the prestimulus fMRI signals in the default-mode network predict the subsequent magnitude of pain ratings, evoked potentials and pain network BOLD response^22^.

In two previous studies we have examined oscillatory activity in the pre-stimulus interval for a constant stimulus at the individual pain threshold^23,24^. For trials rated as painful, significant increases in the theta-(3–7 Hz) and low gamma-(28–32 Hz) band range before stimulus onset were observed. In particular, the oscillatory power within the theta-band before stimulus presentation was closely related to later pain ratings and the origin was located mainly in the insula. The results suggested that the theta-band oscillatory power is related to the differentiation of nociceptive input with respect to the pain domain and therefore reflects the state of sensitivity for the characterization of the stimulus. This result is in accordance with previous data indicating that pain-related responses in the theta frequency range at frontal electrodes code for interindividual variations in the perception of pain^25^. In both studies the rating of a constant stimulus varied across measures to a high degree (in some volunteers about 80% on the VAS for the identical stimulus) which resulted in the perception of different stimuli although the intensity was always identical. This demonstrated that the subjective feeling of a pain intensity as well as the discrimination of the input with respect to the pain domain is affected by several parameters including the pre-stimulus state of neural activity. Therefore, we hypothesize that pre-stimulus theta-band power affected the main level of perceived pain, but also the discrimination of stimuli with different intensities with respect to the feeling of pain. In particular for oscillatory states that precede the stimulus, the correlative nature of such observations does not allow strong conclusions about causal relations. To test the functional relevance of ongoing theta-band power within the insula for pain discrimination more directly, we here tested the modulation of ongoing activity using an EEG based brain–computer interface (BCI). Two groups of volunteers were trained to modulate the theta-band power located in the insula in opposite directions (increase or decrease of power), while the training signal of the BCI was displayed in a consistent way across groups (see methods). This way, each group served as the control group for the other one, and the task characteristics were kept constant for all participants. Further, conditions were blinded for the researchers interacting with the volunteers. The developed BCI processed the EEG signals online including artefact check and source localisation, assuring a feedback signal based on neural power within the right insula (contralateral to the stimulation site). The aim of the BCI was to modulate the oscillatory state of insular based theta-band power across six training sessions. To test whether this modulated oscillatory state affected not only the main level of pain but also the pain discrimination, nociceptive processing at six different intensities was examined at each training and volunteers were asked to indicate the intensity on a pain scale. The stimulus intensities were determined by the individual pain threshold to examine the sensitivity of the system for the differentiation of nociceptive input with respect to the pain domain. The consequences on pain processing were also examined at the neural level by the evaluation of stimulus evoked theta-band power.

## Results

### Modulation of power across training

**Figure 1:**
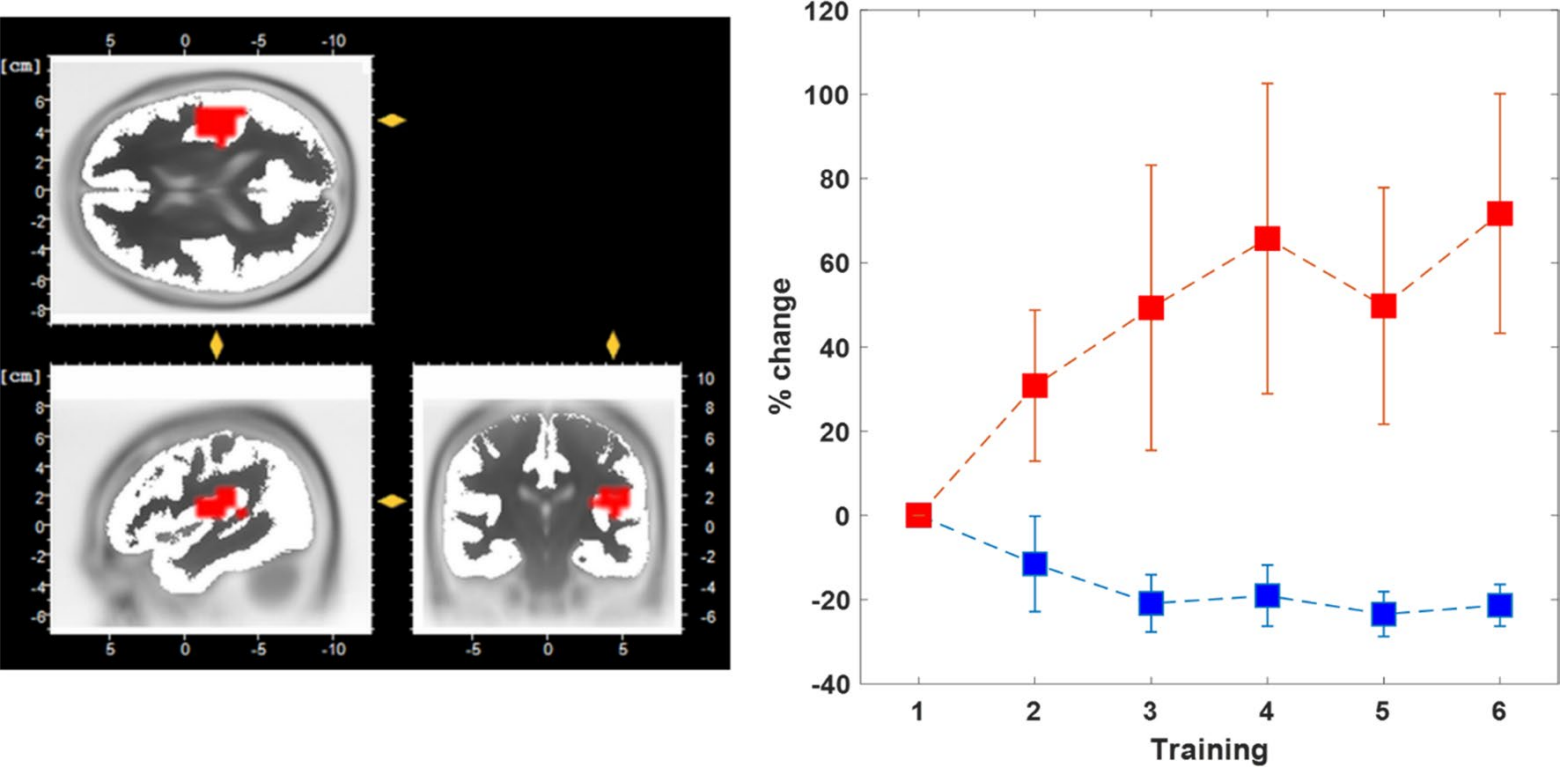
Region of interest in the right insula used to extract theta-band power online (left). Change of insula source space EEG power in theta-band (3–7 Hz) for up-training (red) and down-training (blue) groups (right). Depicted are percent change across training sessions 2–6 relative to the first training session. Error bars depict ± 1 standard error of the mean (SEM, n = 10 in every group).

Both groups showed differential modulation of insular based theta-band oscillatory power across the six training sessions. In both groups about two thirds of the participants (10 up, 10 down) successfully attained control over the theta-band power in the insular region of interest (ROI) and modulated the power in the desired direction (see Fig. 1). A repeated measures ANOVA (factors: training group and session) shows a significant interaction for training group (up/down) and the training session (*p* = 0.019, *F*(1,5) = 2.8) as well as a significant main effect for group (*p* < 0.01, *F*(1,10) = 8.4). Separate *t* tests for the up and down group revealed that power was modulated significantly from the first to the last training session for both directions (up: *p* = 0.032, *t(9)* = 2.52, down: *p* = 0.02, *t(9)* = − 4.3). Therefore, in both groups ongoing theta-band power was succesfully modulated and the consequences for pain processing could be examined.

### Rating

Pain processing was assessed at each training day indepedently from the training before and after the training session. Importantly, the pre-training pain processing is not directly affected by the BCI training on the same day and therefore is able to track stable long-term changes in the pain processing. The overall mean rating was not different between groups (repated measure ANOVA with factors: training group and session, main effect of group F(1,9) = 0.18, *p* = 0.68,) and did not change with training session (F(1,5) = 0.36,* p* = 0.88; interaction: F(1,5) = 0.59, *p* = 0.71).

**Figure 2:**
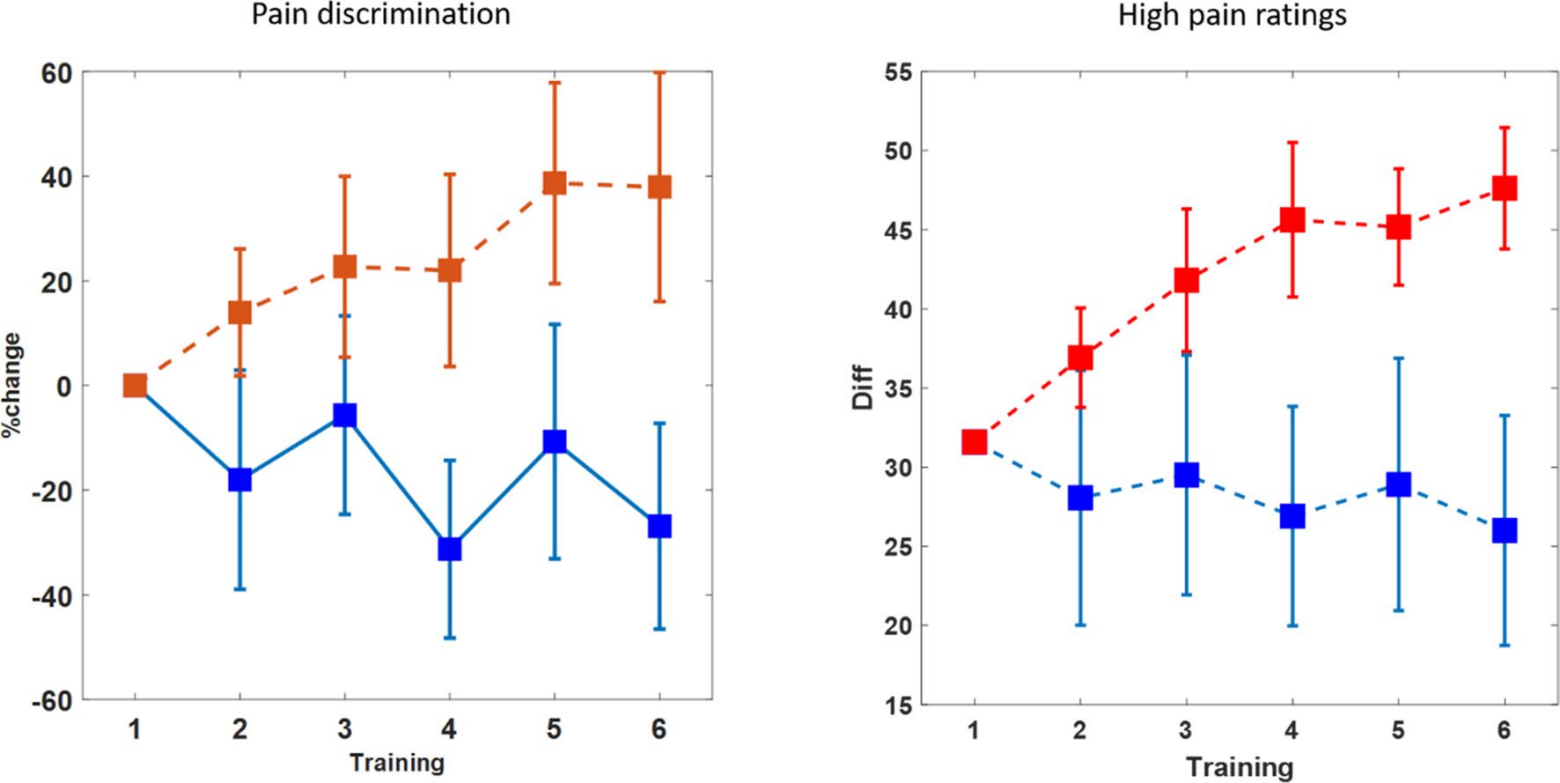
Pain ratings for each training day were assessed at six different intensities in the pre-training period. The discrimination between the different intensities was estimated as the distance of the ratings and reflects the modulation of the sensitivity of the pain system (left). The increasing sensitivity in the up-training group and the decreasing sensitivity of the down-training group is also reflected in the ratings for the higher painful stimuli (right, here the difference to the minimum rating is shown). Error bars depict ± 1 standard error of the mean (SEM, n = 10 in every group).

To estimate the pain discrimination of the different intensities, the differences of the ratings between concsecutive intensity levels were calculated (i.e. rating for level 6 minus rating for level 5 ect.). The sum of this differences reflects the differentation of nociceptive input with respect to the pain domain. For the rating from the pain measurment diretcly preceeding the BCI training, this measure was modulated differentiatly between groups, demonstrating an increase sensitivity in the up-training group and a decrease for the down-training (see Fig. 2). A repeated measures ANOVA (factors: training group and session) shows a significant interaction for training group (up/down) and the training session (*F*(1,5) = 2.4, *p* = 0.04) as well as a significant main effect of the training session (F(1,5) = 2.4, *p* = 0.04,), but no general difference of group (F(1,9) = 3.5,* p* = 0.07). Further, a correlation of the individual pain discrimination index (sum of rating differences) with the change of insular theta-band power during training showed a marginal relation between the training and the change in the rated pain discrimination (r = 0.18, *p* = 0.049).

This effect was explored in more detail by limiting the analyses to the trials that were rated as higher painful (above 50 on the rating scale, Fig. 2 right). The processing of this trials can be assumed to be dominated by the processing of pain whereas the discrimination of the lower ratings might be more related to sensory aspects. For this high rated trials the distance of the rating to the minimum rating also increased across training for the up-training group compared to the down-group (repeated measures ANOVA (factors: training group and session, interaction: F(1,5) = 3.5, *p* = 0.006, main effect training session: F(1,5) = 4.4, *p* = 0.001, main effect training group: n.s.). Separate *t* tests revealed a reliable increase for the up-training group (t(9) = 4.2, *p* = 0.002), but no reliable decrease in the down-group (t(9) = 0.7, n.s.).

**Figure 3:**
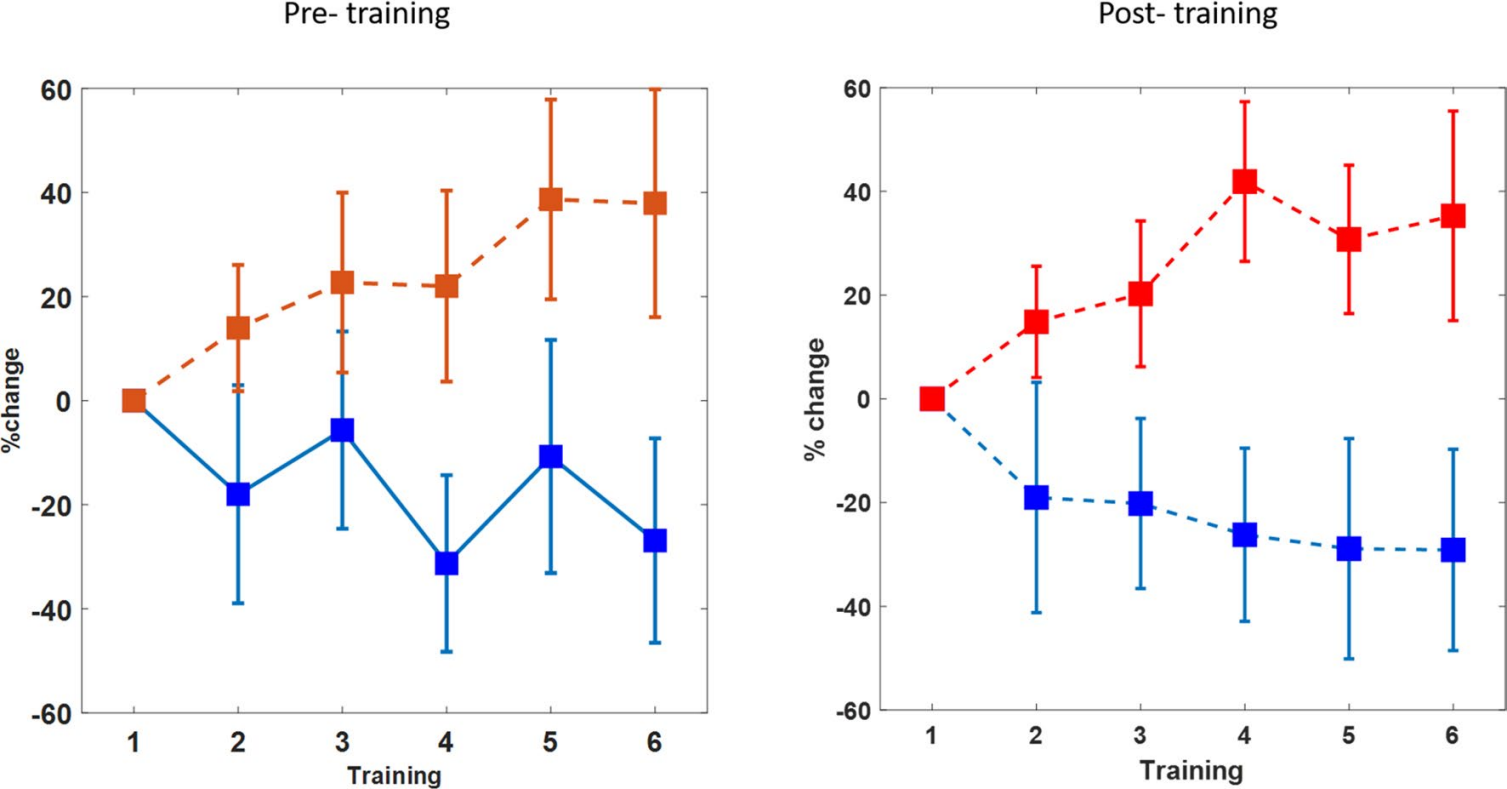
Comparing the pain discrimination between the pre-and post-training period (pre-training presented left and is a repetition from Fig. 2, but here directly compared to the post-training (right panel)). The differentiation between the groups was slightly enhance in the measure after each training, but not reliably different to the pre-training measure indicating the induction of longer lasting effects by the training. Error bars depict ± 1 standard error of the mean (SEM, n = 10 in every group).

Comparable results were obtained from the pain measurment diretcly after the BCI training (see Fig. 3). The pain discrimination was modulated differntially between groups (main effect of training session: *F*(1,5) = 3.3, *p* = 0.008, main effect of training group: *F(1,9)* = 5.9, *p* = 0.03, and interaction of group × session: *F*(1,5) = 3.3,* p* = 0.01). To estimate possible differences in the pre- and post-training measurements both measures were compared statistally and the repeated measurment ANOVA (with factors training group, session and pre-or post training) did not show a reliable difference (interaction of training group × session × pre-post F(1,5) = 0.4, *p* = 0.8) indicating a comparable modulation of pain sensitivity in both paramters (interaction training group x session F(1,5) = 2.9, *p* = 0.02). On a descripitve level the post-training results show a clearer dissociation between groups early in training and the difference appeared latter in training for the pre-training measurement. This could be expected but the small difference between both measure is indicative for a more stable effect on the pain sensitivity that lasted across different training days.

### Pain related theta band power

**Figure 4:**
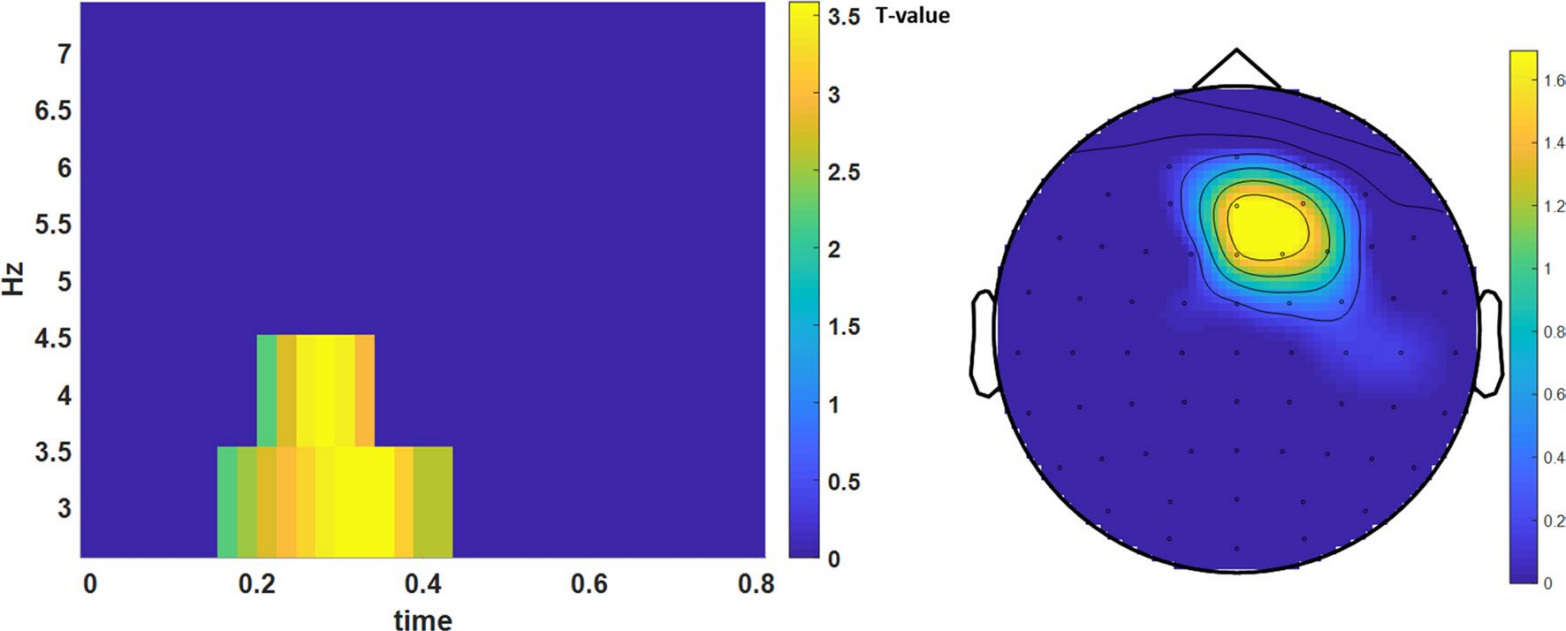
Pain evoked theta-band power measured before the first and the last training. Time-frequency resolved statistical (t-) values were shown at the maximum electrode AFz (left) and indicated different modulations of theta-band power across training between the two groups (only significant values after correction for multiple comparisons were shown). The interaction effect indicated an increase of pain evoked theta-band power in the group that trained to increase theta-band in the independent sessions around 300 ms after stimulus onset (0) and a decrease in the other group. In the right panel the topography of this effect is shown (n = 10 in every group).

To test for a transfer of modulations of oscillatory power during BCI training to neural stimulus processing, pain stimuli were presented without a BCI feedback in a separate session (before and after the actual BCI training). EEG data was recorded and analysed to detect training related changes in pain processing within the theta-band at the first and the last training. EEG was transformed in the time–frequency domain within the theta-band and analysed to detect different modulation across time and group. Therefore, the differences between the first and last training were computed first and then compared between groups. The results for the most important pre-training measure demonstrated a significant interaction effect (training group x session) over frontal electrodes with a maximum at electrode AFz for the measurement before as well as after the training in the frequency range from 3 to 5 Hz (corrected for multiple comparisons) (Fig. 4).

**Figure 5:**
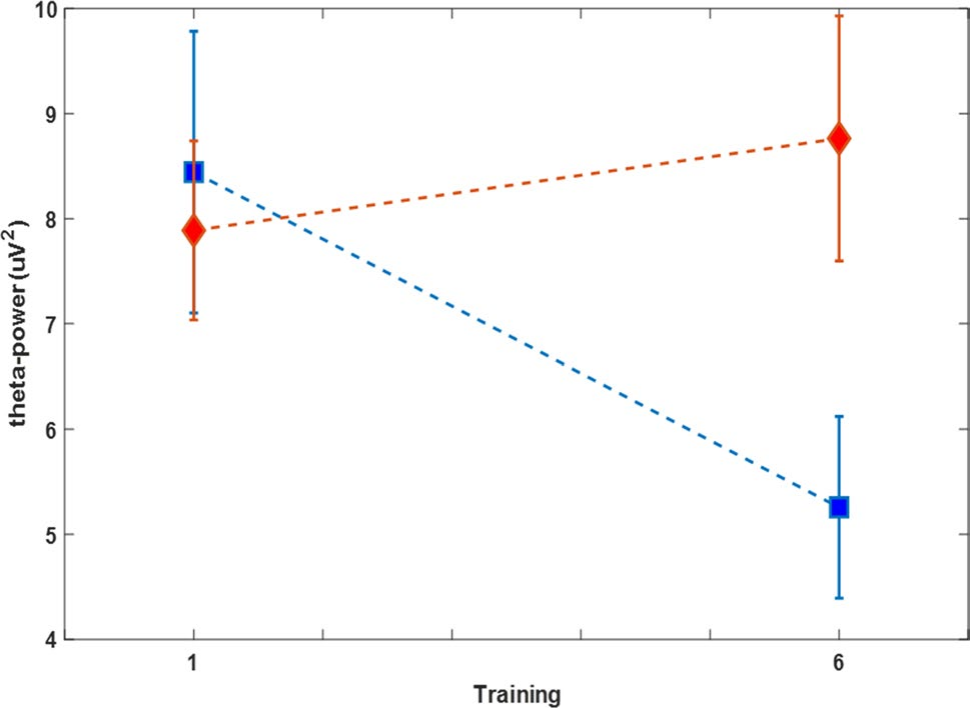
Pain evoked theta-band power. Mean power from 200 to 350 ms (3–5 Hz) measure at before the first and before the last BCI-training. The interaction effect shows an increase of pain evoked theta-band power in the group that was trained to increase ongoing theta-band power (red) and a decrease in the down-group (blue). Error bars depict ± 1 standard error of the mean (SEM, n = 10 in each group).

The interaction effect was additionally tested in more detail using a repeated measures ANOVA (factors: training group and session) by extracting the mean theta band power from 200 to 350 ms at the maximum electrode AFz (from 3 to 5 Hz). The results of the measurement before the training demonstrated a significant interaction of training group (up/down) and training session (*F*(1,5) = 9.1, *p* = 0.007) indicating an increase of pain evoked theta-band in the up-group and a decrease in the down-group (Fig. 5). A comparable effect was detected in the theta band measured directly after the training (post-training). Again a reliable interaction of training group (up/down) and training session (*F*(1,5) = 4.5, *p* = 0.04) indicated a differential modulation of the theta band power. Separate*t* tests revealed a reliable decrease for the down-group (pre-training: t(9) = 3.7, *p* = 0.005) but no significant increase for the up-group.

## Discussion

Here, we demonstrated a non-invasive method to modulate insular oscillatory activity within the theta-band. By assigning the participants to two training groups, we demonstrated that the modulation can elicit an increase as well as a decrease in power. Importantly, the induced modulation of an ongoing oscillatory state shows a transfer to independently assessed pain processing and there affected post-stimulus processing on the neural as well as on one distinct aspect of behavioural outcomes. While the main level of perceived pain was not affected by the training, the discrimination between the different levels of pain were modulated differentially in both groups. The dissociable effects in both groups are indicative for a functional role of the state of theta-band power for the discrimination of nociceptive input with respect to the pain assignment.

Successful decoding of subjective pain intensity involves aggregating data from many distinct brain regions^2,26^. Previous studies indicated the insular cortex is an integral part of the pain processing network although the function is not exclusively pain specific^27^, but contribute important partial information on the magnitude of the resulting percept^2,17^. Further, a functional relevance for pain and pain-related expectation effects is discussed for evoked theta (3–7 Hz)^25^, alpha (8–12 Hz)^28^ and higher gamma (around 60–90 Hz) band power^29^ in relation to different function for translating a sensory input into a pain precept. In particular, for theta-band power a close correlation with the subjective pain experience has been reported^25^. Besides these effects reported for post-stimulus processing, the relevance of pre-stimulus theta-band power has been demonstrated recently^23,24^. The previous results indicated that the function of insular based theta-band oscillations may be related to translating sensory input into a pain percept and discriminating sensory input with respect to the pain domain. The functional relevance of an oscillatory state can be tested more directly by modulating this oscillatory state and testing for an effect on the discriminatory ability of the system. With the specifically designed BCI we were able to modulate the oscillatory power in the two desired directions. Therefore, the BCI can be regarded as a non-invasive tool to modulate an oscillatory state in a circumscribed brain region. The advantage of this method has been demonstrated in previous studies on memory and perception^30–32^. In the pain domain, one study used a real-time fMRI signal to modulate activity within the rostral anterior cingulate cortex and could demonstrate corresponding effects on the pain processing in healthy volunteers as well as in pain patients^33^. The results of the present study show that the majority of volunteers were able to modulate the neural signal in the given direction, even though some of the participants were not able to regulate their oscillatory power in accordance with the experimental demands. Such non-responders are mentioned in the literature, with an estimated share of around 30% of the participant population^34–36^.

In both groups the oscillatory state during training was modulated as induced by the training signal, demonstrating that a voluntary influence on this signal can be learned. The amount of training success was nominally higher in the group that learned to up-regulate the theta-band power compared to the down-group which may be related to the fact that a value that is estimated during a passive baseline is harder to reduce than to increase. However, in both groups a reliable modulation of insular based theta-band activity was observed. Interestingly, the induced modulations of oscillatory power affected a specific aspect of pain processing measured at each training session. On the behavioral level, six different stimulation intensities were tested and had to be rated with respect to the pain attribute. Against our hypothesis, the overall mean pain level was not affected by the training emphasizing the view that pain is a complex process that consists of several networks that are not affected by our training. The negative result with respect to the general level of the pain ratings may also be related to the power of the study with only 10 participants in each group. Therefore, this effect should be further examined in subsequent studies. However, the discrimination of the different intensities was modulated in close relation to the training direction and can be regarded as a small but important aspect of pain processing. The rating differences between the different intensity steps were increased in the up-group and decreased for the down-group with training and showed a relation to the training success. This modulation of the discriminatory function was most pronounced for trials that were rated as more painful that supported the interpretation that this discrimination is related to the subjective feeling of pain. Interestingly, this behavioral effect was observed before as well as directly after the training session. The effect before the actual training clearly demonstrated, that the BCI training induced longer lasting effects that remained stable across several days. This interpretation is supported by the results of the theta-band power that is evoked by the pain stimulation and is differentially modulated in the two groups. The evoked theta-band power around 200-350 ms after stimulus onset showed an interaction of time by condition However, this interaction was mainly driven by a decrease for the group that down-regulate the ongoing theta-band power although the training effect was more pronounced for the other group. This discrepancy may be related to the fact, that a downregulation from a passive baseline is more difficult to achieve than an increase. Therefore, also a nominal smaller effect of training to reduce ongoing theta-band power could in principle result in a stronger effect during pain processing. Further, it is important to note that no linear additive relation of pre- and post-stimulus power can be assumed^37^. The pain-related post-stimulus theta response is well documented, and reflects a central pain processing step identified as a pain-evoked potential which has been analysed in the time^14,38^ and frequency domain^39^. It was reported that the theta responses reflect rather constant physiological and psychological traits of the individual^25^. Further, a relation of the pain-evoked potential and the individual sensitivity to pain has previously been observed in this time range^40^. The results indicate that the evoked theta-band power can be regarded as the basis for an appraisal process which in turn modulates the subjective pain intensity. Such an interpretation is in line with the notion that the formation of a conscious pain percept occurs around 250–500 ms after stimulus onset^14^. In accordance with this interpretation the different modulations in the two groups of the pain evoked theta response may reflect a stable change in pain processing induced by the modulation of the ongoing theta-band state by the BCI. The effect of an ongoing state on pain processing is well characterized during placebo analgesia which also resulted in decreased oscillatory power in the theta-band^25^. Within this context, pain processing has been formally conceptualized as within a predictive coding framework where the pain percept is critically determined by expectations and their modification through learning^41^. Here, expectations are formulated as top-down processes which affect pain processing via Bayesian integration. A modulated oscillatory neural state can also be regarded as a top-down process affecting the shape of a neural prior.

In summary, we demonstrated that modulation of insular based theta-band power can be realized using a BCI and that the modulated oscillatory state changes important aspects of pain processing both on the neural and behavioural level. This strongly indicates a causal role of ongoing theta-band power for the processing of nociceptive input and in particular for the differential assignment of pain characteristics to nociceptive input. As the subjective pain percept emerges from the processing within a complex network, our approach affects only one—but important—part of this network, thus demonstrating functional relevance. This attribution combined with functional results described here add to the advancing mapping of the pain network and may be valuable for future clinical applications.

## Materials and methods

### Participants

A total of 33 healthy participants (19 female) were recruited to partake in the experiment. The sample size was based on previous neurofeedback studies that revealed reliable effects for a modulation of a specific frequency range with a sample from 8–12 subjects^31,32^. Their age ranged from 21 to 32 years (*M* = 24.7). All participants received monetary compensation of 15 EUR per hour for their participation. In the first session, participants were given information about the training schedule and gave written informed consent. They were then randomly assigned to either the up- or down-training group. All participants were told that they could discontinue their participation at any time and would be compensated for the time spent thus far. Participants with a history of drug abuse, chronic pain conditions or currently being under pain medication were excluded during recruitment. All female participants reported using hormonal contraceptives.

### Procedure

On the first session, the participants were given information about the study protocol, and gave written informed consent for their participation. The study was approved by the ethics committee at the Hamburg Medical Association. Recently, a consensus paper for the design of neurofeedback studies was published^42^ and the corresponding checklist can be found in the Supplementary materials.

**Figure 6:**
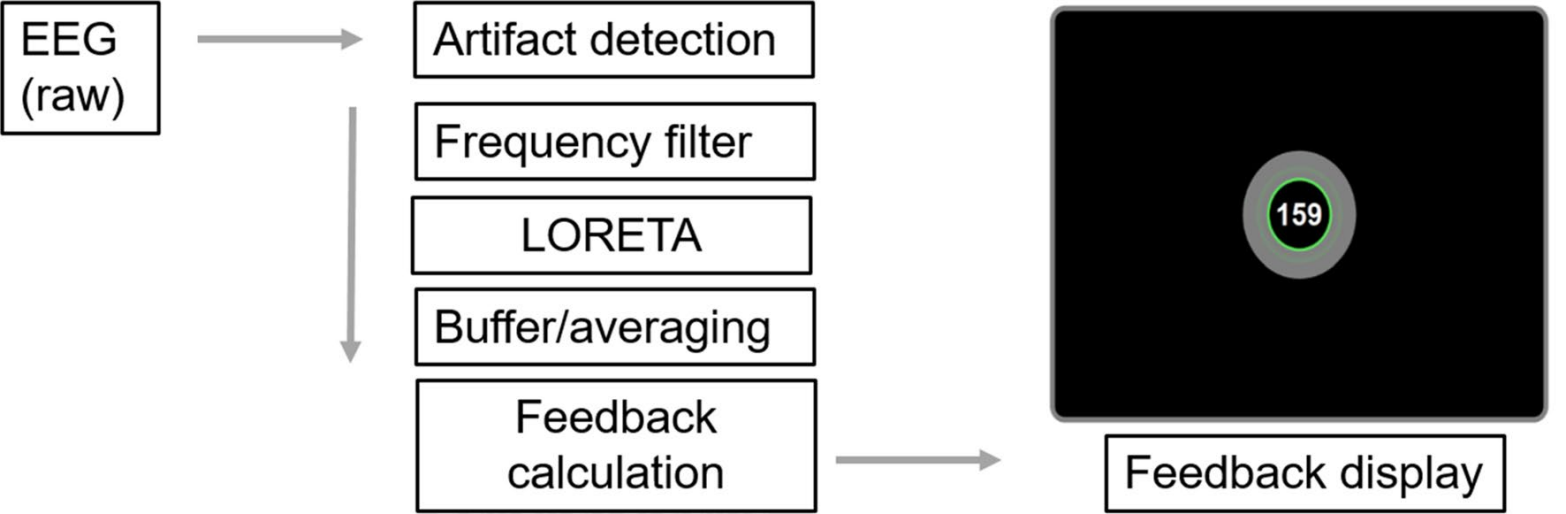
The hierarchical tree of analysis plugins used for the online feedback during the training. As a feedback signal the absolute success measure was presented to the participants as a number on the screen. This signal was identical in both groups and did not indicate whether a participants belonged to the up- or down-group. Therefore, the procedure was blinded to the participants as well as to the instructor. The Feedback display as shown for the participants also included information about the last ten seconds of success as color-coded circles around the feedback value (green circles representing positive success).

Each participant was invited to six sessions over 2–3 weeks. Before the first session the individual pain threshold was estimated (see below). Further, before the actual BCI training, baseline pain stimulation and ratings were conducted. The following BCI training was performed for about one hour at each session. After each BCI training again pain stimulations and rating were assessed. Neural correlates of pain processing were assessed at the first and the last training (see Fig. 6 for an overview).

### Methods

#### EEG

Oscillatory brain power was recorded using an active 64 channel Ag/AgCl electrode system (ActiCap64, BrainProducts, Gilching, Germany). A subset of the 60 most central electrodes within the extended 10–20 electrode system was used to record scalp electrical activity (all impedances kept below 20 kΩ). The remaining four electrodes were used to record a bipolar, bidirectional electrooculogram (EOG). One pair of EOG electrodes was positioned above and below the left eye, the second pair was attached close to the canthi of the eyes. All signals were digitized using two 32 channel BrainAmp amplifiers (BrainProducts, Gilching, Germany) at a sampling rate of 250 Hz. A low cut-off filter was set at 0.53 Hz, a high cut-off at 1000 Hz and a notch filter at 50 Hz mains frequency. Data were recorded to disk using BrainVision Recorder (v1.20.0601, BrainProducts, Gilching, Germany).

#### Feedback system

**Figure 7:**
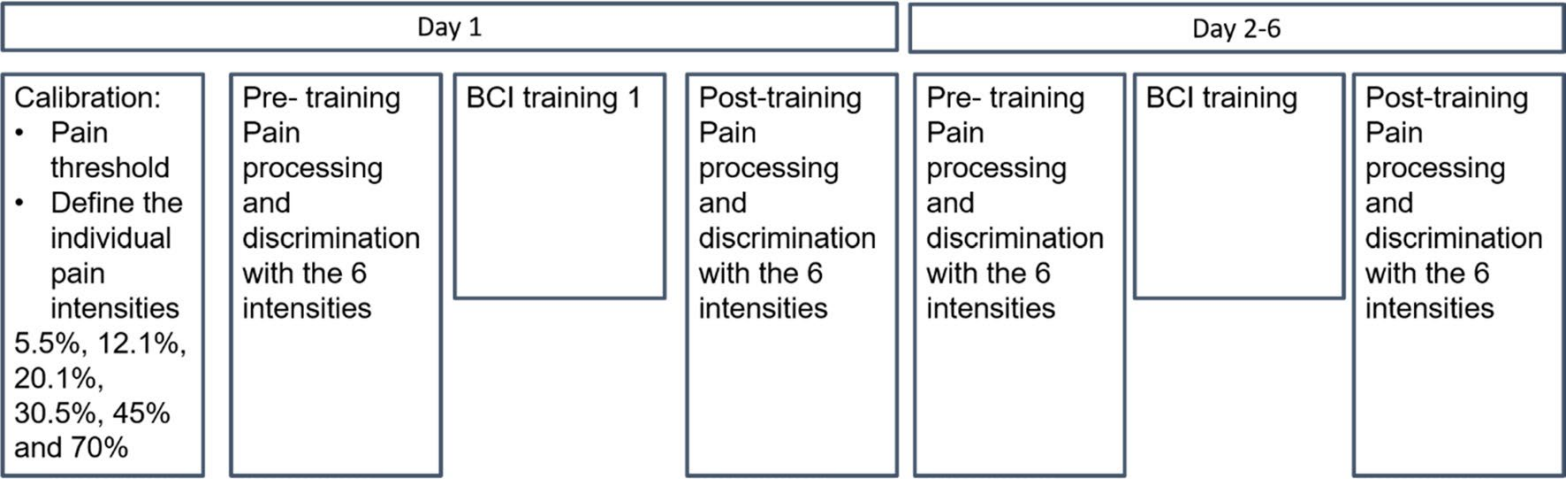
Overview of the experimental design. At the first day the pain intensities were individually calibrated. Before and after each training pain processing and discrimination was assessed.

The recorded EEG data were concurrently streamed over a TCP network socket to a second computer, where it was received in BrainVision RecView (BrainProducts, Gilching, Germany). Within RecView the data were passed along a filter chain, as detailed in Fig. 7.

First, data were submitted to a custom RecView plugin for artefact detection. The plugin was specifically designed to detect eye movements, eye blinks and muscle activity from the absolute and relative signal amplitudes across channels^30^. A trigger signal for artefact occurrence was then passed alongside the EEG data through the filter chain for subsequent use. Theta-band power was extracted using a bandpass filter at 3–7 Hz with a slope of 96 dB/octave. A spatial region of interest (ROI) was defined at the right insular cortex and modelled as a sphere with a radius of 2 cm around the MRI coordinates (− 47, 0, 4, Fig. 1). These coordinates were used with the low resolution electromagnetic tomography (LORETA) method^43^ to derive the summed current density within the ROI for the theta-band signal. The well-established LORETA method operates by finding the maximally smooth current density distribution in source space for a given surface signal configuration^44^. A custom plugin written in C# then collected the resulting data in blocks of 50 samples each, which were aggregated into a ring buffer holding one second of sampling data. The script evaluated the training success for every second of data once the ring buffer had been fully filled. The power was then averaged across all samples within the current second and the mean power value *M*_train_ was compared to an individual baseline value *M*_baseline_ by the means of Z-Scores. The baseline value was estimated at the start of each training session during a 2 min long passive period with eyes open. For the “up” training group the training success was defined as *success* = (*M*_train_ − *M*_baseline_)/*SD*_baseline_ * 100. For the “down” training group, baseline mean and training mean were swapped in the formula, reversing the effective training direction while keeping the feedback consistent across both groups. This success measure was presented to the participants as a number on the screen and the participants were encouraged to increase this number without any given strategy. To provide the participants with a more steady feedback of their short-term performance, the last ten seconds of success were color coded as circles around the feedback value. When the success value was above 0, the circle would show a green color, when it was below 0, the circle would be grey. If the artifact plugin had detected eye movement, the feedback was stopped and an eye symbol appeared in the center of the circle for one second. Training data and information about seconds contaminated by eye activity were recorded to a file for later analysis. The whole data transfer and processing pipeline produces an average delay time of around 50-100 ms. The data from this delay are buffered and saved for the next processing. Thus, the feedback over each past second of theta-band power is given within 50–100 ms. The most time consuming processing step is the calculation of the LORETA reverse solution which is estimated only once in the beginning. Then, the following processing with the already computed transformation matrix is very quick (a few milliseconds).

#### Pain measurements

Pain stimuli were delivered using electrocutaneous stimulation (DS7A Peripheral Stimulator; Digitimer Ltd., Hertfordshire, UK). Electrode position on the abductor/flexor pollicis brevis was determined at half the distance between the first knuckle of the index finger and the first knuckle of the thumb. The stimulator was set to generate a single, monophasic pule of 1 ms length, eliciting a pinprick-like sensation that is comparable to laser stimulation.

##### Pain calibration (first day)

Before the first training sessions started, a calibration procedure was carried out, assessing the individual pain threshold and the subjective 70% pain level and then define the six different intensities that were used at all training sessions. To find suitable starting points for psychophysical estimation, participants were presented with some random stimulus intensities picked by the experimenter, which they should then rate on a 100 point visual analogue (VAS) scale. A high and a low starting point was estimated by linear regression. The low starting point was defined as the threshold intensity between non-pain and pain, the high starting point was defined as 70% pain intensity. The starting intensities were then used as initial estimates for a psychophysical anchoring procedure which robustly estimated individual stimulation levels subsequently. The intensities necessary to elicit comparable ratings across participants were determined using the QUEST algorithm^45^. Per estimated parameter, two estimation processes were run in parallel. One started 20% above the starting point, one 20% below. Both runs consisted of 15 trials each and were randomly intermixed, as to maximize the variance in the stimulus levels tested consecutively. This procedure was repeated for both pain threshold as well as 70% pain level. The final estimates were computed as the mean between the two QUEST runs for each parameter. To assess the discrimination between nociceptive stimuli, a set of six logarithmically spaced intensities were computed, ranging from threshold intensity across 5.5%, 12.1%, 20.1%, 30.5%, 45% and 70% subjective pain level. The subjective stimulation intensities were used throughout the following training session to be able to relate any changes in the average ratings. At each training session a rating was performed to assess changes in subjective pain susceptibility. To this end the previously determined stimulation levels for each participant were presented three times each, totalling to 36 stimulations per rating procedure. Pain was rated on a scale, with a score of 0 corresponding to “no pain” and 100 corresponding to “unbearable pain”.

### Data analysis

#### EEG feedback data analysis

The power data that were recorded online during training (online data) were used to assess the training success per participant. A certain number of participants in training paradigms can be expected to fail to regulate their own brain activity. In current literature it is often reported that about one third of participants will not be able to willingly change their brain activity in a systematic manner^34–36^. Hence, participants that failed to achieve a power difference between the first and last session in the respective training direction were not included in further analyses (difference ≤ 0). This way, the training outcome on the first training day was used as a baseline for all consecutive sessions.

The success measures for the online data were analysed using a repeated measures ANOVA with the factors “training group” (up vs. down) and “session” (1–6). To test, whether the training activity indeed changed from baseline level in both groups, two separate one-sample*t* tests were conducted for the last training session. All statistical test were estimated with matlab (MATLAB Version: 9.9.0.1467703 (R2020b), Natick, Massachusetts: The MathWorks Inc).

#### Pain ratings

The behavioral rating data were segmented into training sessions and means were calculated for the three repeated ratings at each stimulation level. The sensisitvity of the pain system to the different intensities was estimated by caclulating the differences of the ratings between concsecutive intensity levels (i.e. rating for level 6—rating for level 5 ect. + rating for level 5-level 4 etc.). The sum of this differences reflects the differentation of nociceptive input with respect for the pain domain. Further, the sensitivity was explored in more detail by limiting the analyses to the trials that were rated as higher painful (above 50%). For these trials the distance to the minimum rating was estimated in each session. Finally, the overall mean across all intensities was computed for each session. For all measures a reeated measures ANOVA with the factors “training group” (up vs. down) and “session” (1–6) was computed. To test for possible differences in the pre- and post-training measurements both measures were compared statistally and the repeated measurment ANOVA (with factors training group, session and pre-or post training).

#### EEG pain data analysis

For the first and the last training session, EEG data was recorded and analysed to detect training related changes in pain processing in the theta band. This analysis was restricted to the pain trials where stimuli of the three highest intensities were presented to examine pain related processing. For the lower intensities it cannot be assumed that the stimulus is always felt as painful. Therefore, also a pooling across all intensities would result in a mixture of pain processing and simple perception without pain. However, we were only interested in the subjective feeling of pain. To keep the sensory aspects constant that could influence the evoked activity we used all trials from the highest intensities.

Independent component analysis (ICA) was used to remove blink, eye movement, cardiac, and muscle artifacts based on visual inspection of the time course, spectrum, and topography of each component. Further a semi-automatic artefact tagging / rejection method implemented in the fieldtrip toolbox^46^ was used. Hereby, z-scores for individual trials were computed against all segments, and deviations larger than 6 standard deviations were tagged for visual review. This method was used with the parameters of range, absolute value and standard deviation of the signal. Overall, on average 4.5% of EEG segments were rejected. The resulting EEG data were then transformed into the time–frequency domain using a multi-taper method. The frequency range of interest was 3–7 Hz, the time range from 0 to 0.8 s (around stimulus onset) was processed with a time resolution of 0.05 s. The windows size was 0.4 s, frequency smoothing was 2 Hz. To test the interaction effect of group X training the difference time frequency spectra between the first and the last training within each group were computed and the resulting difference was compared statistically between groups. For statistical testing the difference spectra were analysed using non-parametric cluster based permutation tests as implemented in the Fieldtrip toolbox (Maris & Oostenveld, 2007). This method controls for the multiple comparison problem using a multi-step procedure. This correction for multiple comparison was applied for all time points, the 5 different frequencies and all central, frontal and temporal electrodes (number of randomizations: 5000, weighted cluster mass). The maximum effect was observed at electrode AFz in the frequency range from 3 to 5 Hz. Here, the interaction effect was additionally tested using a repeated measures ANOVA (factors: training group and session) by extracting the mean theta band power from 200 to 350 ms and from 3 to 5 Hz.

## Supplementary Information

Supplementary Information

## Data Availability

The datasets generated during and/or analysed during the current study are available from the corresponding author on reasonable request. We confirm that all methods were carried out in accordance with relevant guidelines and regulations and that all experimental protocols were approved by the ethics committee at the Hamburg Medical Association (PV7170).
