# Supplementary material for: The modulation of neural insular activity by a brain computer interface differentially affects pain discrimination
Source: Sci Rep. 2021 May 7;11:9795. doi: 10.1038/s41598-021-89206-3 (PMC8105353; doi:10.1038/s41598-021-89206-3)
Supplement: Supplementary file 1 — Supplementary Information [file 41598_2021_89206_MOESM1_ESM.pdf]

Supplementary Information for: The modulation of neural insular activity by a Brain-Computer Interface differentially affects pain discrimination by Taesler & Rose (Checklist from Ros, T. et al. (2020))

Domain	Item #	Checklist item	Reported on page #
**Pre-experiment**			
	1a	Pre-register experimental protocol and planned analyses	-
	1b	Justify sample size	16
**Control groups**			
	2a	Employ control group(s) or control condition(s)	16
	2b	When leveraging experimental designs where a double-blind is possible, use a double-blind	18
	2c	Blind those who rate the outcomes, and when possible, the statisticians involved	16
	2d	Examine to what extent participants and experimenters remain blinded	-
	2e	In clinical efficacy studies, employ a standard-of-care intervention group as a benchmark for improvement	-
**Control measures**			
	3a	Collect data on psychosocial factors	-
	3b	Report whether participants were provided with a strategy	19
	3c	Report the strategies participants used	-
	3d	Report methods used for online-data processing and artifact correction	18-19
	3e	Report condition and group effects for artifacts	-
**Feedback specifications**			
	4a	Report how the online-feature extraction was defined	18-19
	4b	Report and justify the reinforcement schedule	18-19
	4c	Report the feedback modality and content	18-19
	4d	Collect and report all brain activity variable(s) and/or contrasts used for feedback, as displayed to experimental participants	18-19
	4e	Report the hardware and software used	17-19
**Outcome measures**			
Brain	5a	Report neurofeedback regulation success based on the feedback signal	6
	5b	Plot within-session and between-session regulation blocks of feedback variable(s), as well as pre-to-post resting baselines or contrasts	6
	5c	Statistically compare the experimental condition/group to the control condition(s)/group(s) (not only each group to baseline measures)	5
Behaviour	6a	Include measures of clinical or behavioural significance, defined a priori, and describe whether they were reached	6-9
	6b	Run correlational analyses between regulation success and behavioural outcomes	7
**Data storage**			
	7a	Upload all materials, analysis scripts, code, and raw data used for analyses, as well as final values, to an open access data repository, when feasible	-
